# Identification of Elongation Factor G as the Conserved Cellular Target of Argyrin B

**DOI:** 10.1371/journal.pone.0042657

**Published:** 2012-09-10

**Authors:** Beat Nyfeler, Dominic Hoepfner, Deborah Palestrant, Christina A. Kirby, Lewis Whitehead, Robert Yu, Gejing Deng, Ruth E. Caughlan, Angela L. Woods, Adriana K. Jones, S. Whitney Barnes, John R. Walker, Swann Gaulis, Ervan Hauy, Saskia M. Brachmann, Philipp Krastel, Christian Studer, Ralph Riedl, David Estoppey, Thomas Aust, N. Rao Movva, Zuncai Wang, Michael Salcius, Gregory A. Michaud, Gregory McAllister, Leon O. Murphy, John A. Tallarico, Christopher J. Wilson, Charles R. Dean

**Affiliations:** 1 Developmental and Molecular Pathways, Novartis Institutes for BioMedical Research, Cambridge, Massachussetts, United States of America; 2 Developmental and Molecular Pathways, Novartis Institutes for BioMedical Research, Basel, Switzerland; 3 Center for Proteomic Chemistry, Novartis Institutes for BioMedical Research, Cambridge, Massachussetts, United States of America; 4 Infectious Diseases, Novartis Institutes for BioMedical Research, Emeryville, California, United States of America; 5 Novartis Institute for Functional Genomics, Novartis Institutes for Biomedical Research, San Diego, California, United States of America; 6 Disease Area Oncology, Novartis Institutes for BioMedical Research, Basel, Switzerland; 7 Center for Proteomic Chemistry, Natural Products Unit, Novartis Institutes for BioMedical Research, Basel, Switzerland; 8 Global Discovery Chemistry, Novartis Institutes for Biomedical Research, Cambridge, Massachussetts, United States of America; Institute of Enzymology of the Hungarian Academy of Science, Hungary

## Abstract

Argyrins, produced by myxobacteria and actinomycetes, are cyclic octapeptides with antibacterial and antitumor activity. Here, we identify elongation factor G (EF-G) as the cellular target of argyrin B in bacteria, via resistant mutant selection and whole genome sequencing, biophysical binding studies and crystallography. Argyrin B binds a novel allosteric pocket in EF-G, distinct from the known EF-G inhibitor antibiotic fusidic acid, revealing a new mode of protein synthesis inhibition. In eukaryotic cells, argyrin B was found to target mitochondrial elongation factor G1 (EF-G1), the closest homologue of bacterial EF-G. By blocking mitochondrial translation, argyrin B depletes electron transport components and inhibits the growth of yeast and tumor cells. Further supporting direct inhibition of EF-G1, expression of an argyrin B-binding deficient EF-G1 L693Q variant partially rescued argyrin B-sensitivity in tumor cells. In summary, we show that argyrin B is an antibacterial and cytotoxic agent that inhibits the evolutionarily conserved target EF-G, blocking protein synthesis in bacteria and mitochondrial translation in yeast and mammalian cells.

## Introduction

Natural products constitute a major resource for the identification of bioactive molecules. Indeed, most antibacterials in current use are natural products or semisynthetic derivatives thereof. The argyrins are natural peptides produced by myxobacteria and actinomycetes that have an intriguing antibacterial spectrum of activity [1], [2], [3]. This includes the intrinsically drug resistant organism *Pseudomonas aeruginosa*, but not other Gram negatives tested, such as *Escherichia coli* or *Salmonella typhimurium*, unless the cells are compromised in their outer membrane permeability barrier, presumably allowing access to the intracellular target [1], [2]. It has been shown that argyrins inhibit bacterial protein synthesis [2], but the specific cellular efficacy target of these antibacterials has not been identified. Argyrins were also shown to be immunosuppressive [1], [4], [5] and anti-tumorigenic, with more recent investigations providing evidence that argyrin A inhibits the proteasome, induces apoptosis, and blocks angiogenesis by a p27-dependent mechanism [6], [7], [8], [9]. To shed more light on the cellular target of this interesting class of natural products, we employed bacterial and yeast mutant selection and whole genome sequencing to identify the target of argyrin B and explored whether the mechanism of action is conserved in mammalian cells.

## Results

### Argyrin B inhibits bacterial elongation factor G

We first confirmed the activity of argyrin B against *P. aeruginosa* PAO1 strain K767, and also observed activity against another intrinsically drug resistant Gram-negative pathogen, *Burkholderia multivorans* (**Table 1**). *P. aeruginosa* mutants with decreased susceptibility to argyrin B were selected at a frequency of circa 5×10^−8^ on solid medium containing 128 µg/ml argyrin B (minimum inhibitory concentration (MIC), 4–8 µg/ml). Sequencing the genome of one mutant identified a single mutation in the *fusA1* gene, resulting in an amino acid substitution in elongation factor G (EF-G). EF-G mediates the translocation of mRNA and tRNA through the ribosome and is essential for protein synthesis [10], [11]. The *fusA1* gene from 5 additional mutants was then sequenced and found to contain point mutations encoding amino acid substitutions. The overall list of individual substitutions identified in EF-G were: P414S, S417L, S459F, P486S, T671A and Y683C (mutants CDR0052, CDR0054, CDR0055, CDR0056, CDR0057, CDR0058; **Table 1**). Argyrin B had variable solubility in MIC assays (visible precipitate above 16 µg/ml), so a more sensitive strain was used to more accurately determine the change in susceptibility conferred by resistance mutations. *P. aeruginosa* strain Z61 is hypersusceptible to a wide range of antibiotics, due to mutations affecting cell permeability [12], [13]. Strain Z61 plated on 2 µg/ml argyrin B (MIC 0.125–0.25 µg/ml), yielded mutants also having the S417L and S459F substitutions, and identified an additional L663Q alteration (mutants CDA0055, CDA0056, CDA0061; **Table 1**). While mutants with S417L and S459F substitutions were still partially sensitive, L663Q conferred a higher level of resistance to argyrin B (MIC>128 µg/ml, **Table 1**). This suggested that argyrin B was inhibiting bacterial growth by targeting EF-G. *P. aeruginosa* has two similar genes encoding elongation factor G proteins, *fusA1* and *fusA2* (designated here as EF-G1 or EF-G2). All mutations were found in *fusA1*, consistent with the observation that *fusA1* is highly transcribed whereas *fusA2* is not (genechip analysis, data not shown). In *B. multivorans*, argyrin B resistant mutants (frequency circa 10^−9^–10^−8^) also had mutations in *fusA1*, mirroring the scenario observed for *P. aeruginosa* (**Table 1**). To exclude the possibility that selection of *fusA* mutations using argyrin B might reflect an indirect resistance mechanism rather than direct interaction of argyrin with this target protein, we tested if purified *P. aeruginosa* EF-G1 binds to argyrin B. Very tight binding was observed as measured with isothermal titration calorimetry (173 nM; stoichiometry of 1) as well as surface plasmon resonance (SPR, 176 nM). In contrast, EF-G1 S459F failed to bind argyrin B sufficiently to generate a K_d_ value by SPR (data not shown).

**Table 1:** Susceptibility of representative bacteria and resistant mutants to argyrin B.

Strain	Relevant characteristic	Susceptibility to argyrin B (µg/ml)*	Source or reference
*P. aeruginosa*			
K767	PAO1, prototroph	8	[34]
CDR0058	K767, FusA1_P414S_	>128	This study^a^
CDR0055	K767, FusA1_S417L_	>128	This study^a^
CDR0054	K767, FusA1_S459F_	>128	This study^a^
CDR0057	K767, FusA1_P486S_	>128	This study^a^
CDR0052	K767, FusA1_T671A_	>128	This study^a^
CDR0056	K767, FusA1_Y683C_	>128	This study^a^
ATCC 12055	Parent of ATCC 35151	8	ATCC
ATCC 35151 (Z61)	Hypersensitive	0.125–0.25	ATCC
CDA0055	Z61, FusA1_S417L_	64	This study^b^
CDA0056	Z61, FusA1_S459F_	8	This study^b^
CDA0061	Z61, FusA_L663Q_	>128	This study^b^
*B. multivorans*			
NB49004	Clinical isolate	1	I. Chopra
CDA0079	FusA1_P484R_	>32	This study^c^
CDA0080	FusA1_S415L_	>32	This study^c^
CDA0093	FusA1_S415W_	>32	This study^d^
CDA0094	FusA1_L656P_	>32	This study^d^

Susceptibility determinations were conducted using the broth microdilution protocol as described previously [26].

* Argyrin B was not uniformly soluble and occasionally a small amount of precipitate was visible at concentrations greater than 16–32 µg/ml; therefore values here are reported as susceptibility rather than MIC. Selected on 128^a^, 2^b^, 4^c^, or 16^d^ µg/ml argyrin B in solid Mueller-Hinton agar.

### Argyrin B binding to EF-G reveals a new allosteric binding site and novel mode of inhibition

**Figure 1:**
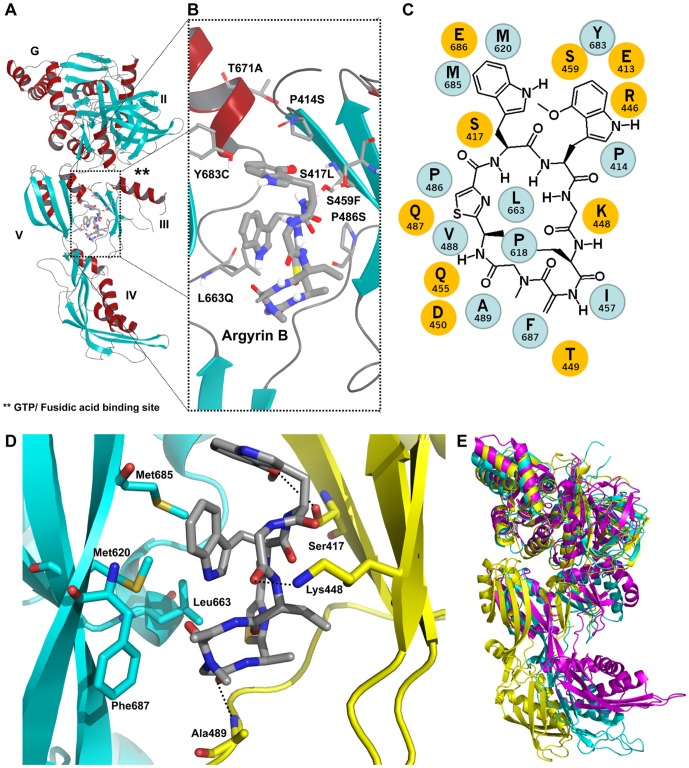
Co-crystal structure of argyrin B bound to *P.aeruginosa* EF-G1. (**A**) The argyrin B binding pocket localizes to the flexible interface between domains III and V, distinct from the GTP/fusidic acid binding domain (**). (**B**) Inset view. (**C**) 2D protein-ligand interaction plot showing the chemical structure of the argyrin B macrocyclic polypeptide and the hydrophobic (cyan) and hydrophilic (yellow) amino-acid residues in EF-G1 which are in binding contact. (**D**) Interactions between *P. aeruginosa* EF-G (domain III in yellow and domain V in cyan) and argyrin B (gray). (**E**) Superposition of Thermus thermophilus EF-G in complex with GTP (magenta), Thermus thermophilus EF-G in complex with the ribosome (ribosome not shown) and fuscidic acid (cyan), and structure of the argyrin B-bound Pseudomonas aeroginosa EF-G (FusA1) (yellow). Superposition was done using domains I and II of each of the protein structures.

Since argyrin B clearly bound to the *P. aeruginosa* EF-G protein encoded by *fusA1*, we sought to derive more detailed information regarding the target binding pocket and possible mode of inhibition. The structure of *P. aeruginosa* EF-G1 in complex with argyrin B was therefore determined to 2.9 Å. The overall domain structure of EF-G1 seen here was similar to that of previously reported bacterial EF-G protein structures [14], [15], [16], [17]. Argyrin B bound at the interface of domains III and V, revealing a novel inhibitor binding site that is clearly distinct from that of the characterized EF-G inhibitor fusidic acid (**Figure 1A**). Key binding interactions between argyrin B and domain III of EF-G1 are defined by hydrogen bonding interactions between the backbone amide of Ala489 and the N-methylated glycine of argyrin B, the hydroxyl group of Ser417 to the oxygen within the methoxytryptophan, as well as from the side chain of Lys448 to the glycine of argyrin B (**Figure 1B, C and D**). Van der Waals interactions are also observed between the thiazole within the argyrin B structure and the backbone of Gln487 and Val488. Interactions between argyrin B and domain V of EF-G1 are defined by van der Waals interactions between the indole ring of the tryptophan portion of argyrin B and Met620 and Met685, as well as additional interactions between argyrin B to both Leu663 and Phe687 (**Figure 1B, C and D**). Amino acid substitutions important for *P. aeruginosa* resistance are shown on the co-crystal structure and clearly line the binding pocket of argyrin B (**Figure 1B**). Of particular interest, the argyrin B-bound protein displays a rotation of domains III and V relative to domains I and II as compared to previously determined structures [15], [17]. A ratcheting of domain IV is observed, indicating that argyrin B-bound EF-G1 adopts a more elongated conformation than has been described previously for *Thermus thermophilus* EF-G in complex with GTP [15] or in complex with fusidic acid and the ribosome [17] (**Figure 1E**). These structural data indicate that argyrin B directly binds to EF-G at a novel allosteric pocket causing EF-G to adopt an extended conformation that is unlikely to be compatible with ribosome binding.

### Argyrin B targets mitochondrial elongation factor G in yeast

**Figure 2:**
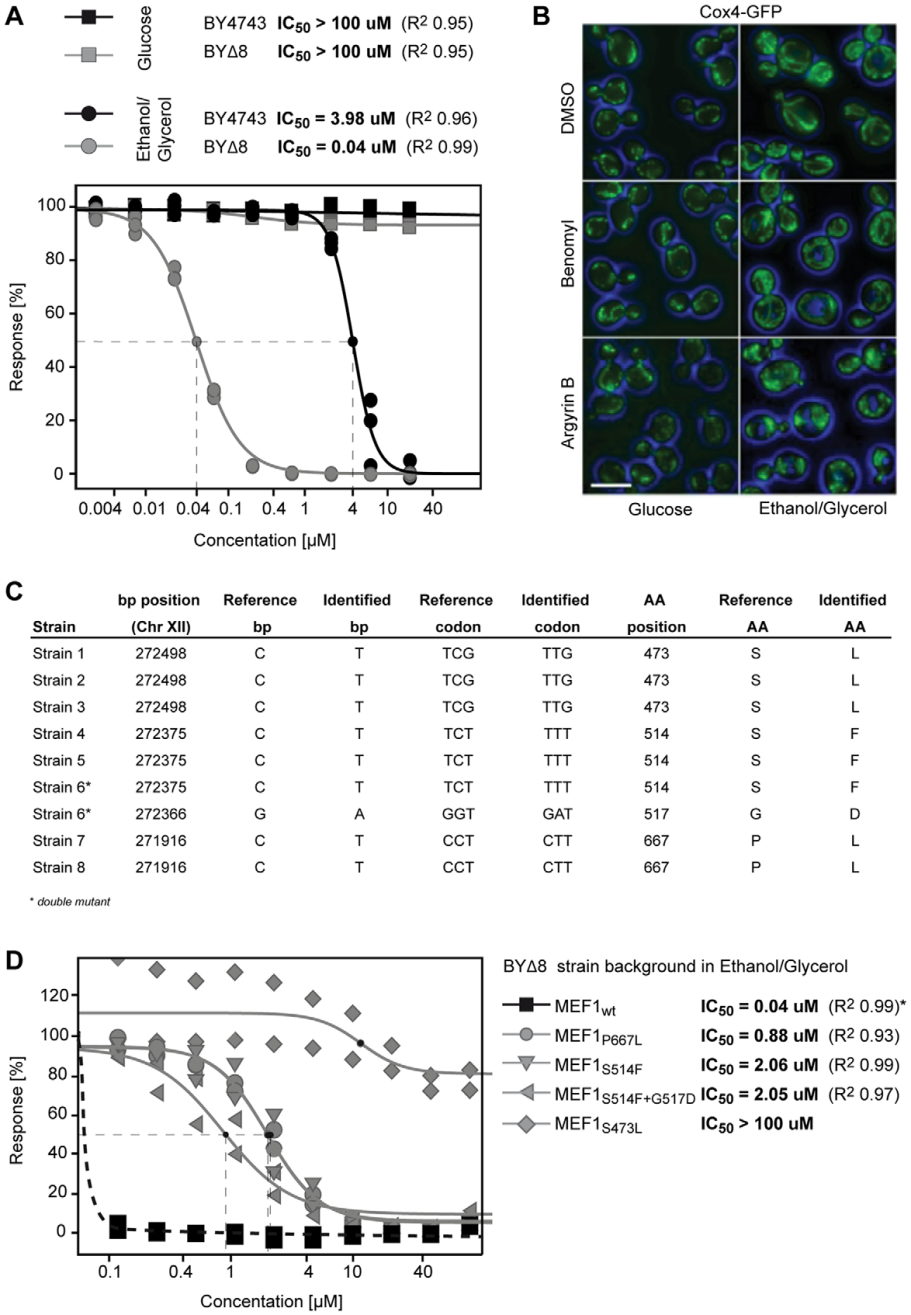
Identification of mEF-G1 as the efficacy target of argyrin B in *S. cerevisiae*. (**A**) IC_50_ curves of argyrin B on wild-type cells (black) and cells deleted for 8 components involved in drug resistance (grey) in glucose (sqares) and ethanol/glycerol (circles). Cells were tested in triplicates, calculated IC_50_ values are indicated. (**B**) Microscopic analysis of Cox4-GFP labeled cells grown in glucose (left panel) and ethanol/glycerol (right panel). Cells were treated with 3 times the IC_30_ concentrations of argyrin B (10 µM on YPEG, 200 µM on YPD) or Benomyl (90 µM) for 30 minutes prior to analysis. Size bar represents 5 µm. (**C**) Sequence analysis of argyrin B-resistant yeast colonies identifies 4 individual base pair changes that all result in amino-acid changes. (**D**) Validation of the identified mutations by introduction of the individual base-pair changes into wild-type cells and recording IC_50_ curves in duplicates. *Except for the DMSO control, all argyrin B concentrations in the range tested resulted in full inhibiton of the wild-type strain and the IC_50_ value from panel A is depicted.

Argyrins are also active against eukaryotic cells [6], [7], [8], [9]. To explore this further, we first determined toxic effects on yeast, which represent another genetically tractable system for mechanistic studies [18]. Argyrin B was inactive against wild-type yeast (BY4741) and a strain deleted for eight genes involved in efflux (strain CMB970, designated as BYΔ8) when tested in rich medium containing glucose as carbon source. However, with ethanol/glycerol substituted as carbon source, argyrin B scored an IC_50_ concentration of 4 µM on wild-type and 0.04 µM on the drug efflux-deleted strain BYΔ8 (**Figure 2A**). While glucose can be metabolized in yeast cells by anaerobic fermentation, energy generation from ethanol/glycerol is strictly dependent on oxidative phosphorylation, suggesting that argyrin B interfered with mitochondrial function. The observed IC_50_ shift in the drug-efflux compromised strain also indicated that argyrin B was a substrate for drug efflux pumps in yeast. Consistent with argyrin B interference with mitochondria, incubating a strain with Cox4-GFP labeled mitochondria [19] in 3× the IC_30_ concentration of argyrin B for 30 minutes followed by fluorescence microscopy analysis showed mitochondrial morphology defects (fragmentation) for cells grown on ethanol/glycerol but not glucose (**Figure 2B**). Treatment with the microtubule toxin benomyl at 3× its IC_30_ had no effect on mitochondria, indicating that the response to argyrin B was not a general stress response.

To identify the specific target of argyrin B in yeast, a mutagenesis strategy [20] similar to that described above for bacteria was used. On ethanol/glycerol medium, argyrin B abolished colony formation of the efflux-defective yeast strain at 0.1 µM. We therefore plated 10^7^ chemically mutagenized yeast cells on this selection medium and analyzed resistant survivors by whole genome sequencing. The *MEF1* gene encoding mitochondrial elongation factor 1 (mEF-G1) was a hot spot for mutations. Four individual mutations were identified resulting in four distinct amino acid changes (S473L, S514F, G517D, P667L, **Figure 2C**). Each mutation was introduced into a fresh BYΔ8 strain background, and these all showed a significantly shifted susceptibility (**Figure 2D**) confirming that the mutations selected in *MEF1* mediated resistance to argyrin B. The A514F/G517D double mutant (Strain 6, Figure 1C) was equally susceptible to the A514F single mutant, suggesting that G517D is likely a passenger mutation not contributing to argyrin B resistance. mEF-G1 is the closest homologue of bacterial EF-G in the yeast *S. cerevisiae*, which supports conservation of the argyrin B target in eukaryotic cells. While P667L represents a novel mutation, S473L and S514F correspond to *P. areuginosa* EF-G1 mutations S417L and S459F, respectively (**Table 2**).

**Table 2:** Key residues involved in resistance to argyrin B are conserved in EF-G homologues.

Organism		Amino Acids in *P. aeruginosa* EF-G1								Homology to *P. aeruginosa* EF-G1 (%)
		414	417	459	486	618	663	671	683	
*P. aeruginosa*	EF-G1	P	S	S	P	P	L	T	Y	100
*B. multivorans*	EF-G1	P	S	S	P	P	L	T	Y	67
*S. cerevisiae*	mEF-G1	**A**	S	S	P	P	L	T	**F**	41
	mEF-G2	P	**G**	**N**	**L**	P	L	**N**	**F**	31
	EF-2	P	**Q**	**A**	P	P	**V**	**G**	**P**	25
Human	mEF-G1	P	S	S	P	P	L	T	Y	40
	mEF-G2	P	**F**	**C**	**L**	P	L	T	**F**	38
	EF-2	P	**R**	**A**	P	P	**V**	**A**	**P**	24
*P. aeruginosa* mutants		S	L	F	S	-	Q	A	C	
*B. multivorans* mutants		-	L/W	-	R	-	P	-	-	
*S. cervisiae* mutants		-	L	F	-	L	-	-	-	

The amino acid sequence of *P. aeruginosa* EF-G1 was used to identify EF-G homologues in the indicated organisms by a BLAST search. EF-G sequences were then aligned using ClustalW, and residues conferring resistance to argyrin B are shown in bold.

### Argyrin B targets mitochondrial elongation factor G in mammalian cells

**Figure 3:**
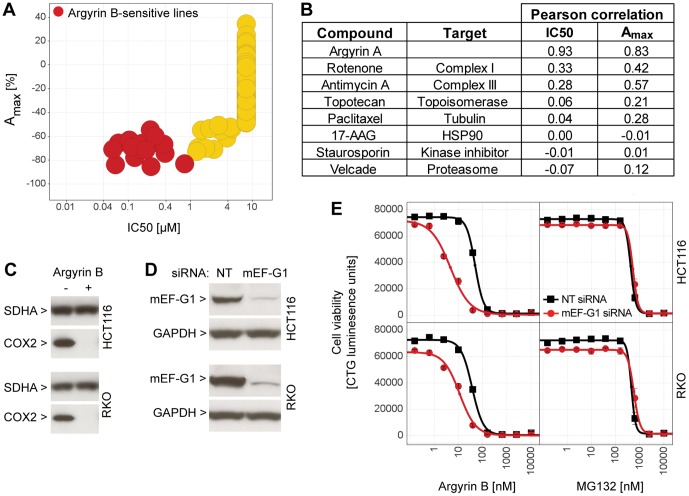
The mode of action of argyrin B is conserved in mammalian cells. (**A**) Cytotoxicity profile of argyrin B across 512 mammalian cell lines showing reduced cell viability with an IC_50_ below 1 µM in 18 cell lines (red). (**B**) Susceptibility to argyrin B (IC_50_ and A_max_ values) was compared to different cytotoxic agents across the cell line panel by calculating Pearson correlation values. (**C**) RKO and HCT116 cells were treated for 4 days with 1 µM argyrin B, and total proteins were extracted and analyzed by immunoblotting for SDHA and COX2. (**D**) Cells were transfected with non-targeting (NT) or GFM1 (encoding mEF-G1) siRNA for 7 days, and total proteins were extracted and analyzed by immunoblotting for mEF-G1 and GAPDH. (**E**) siRNA-transfected cells were treated for 7 days with increasing doses of Argyrin B or MG132, and cell viability was assessed using CellTiter Glo. A representative example of three independent experiments is shown.

**Figure 4:**
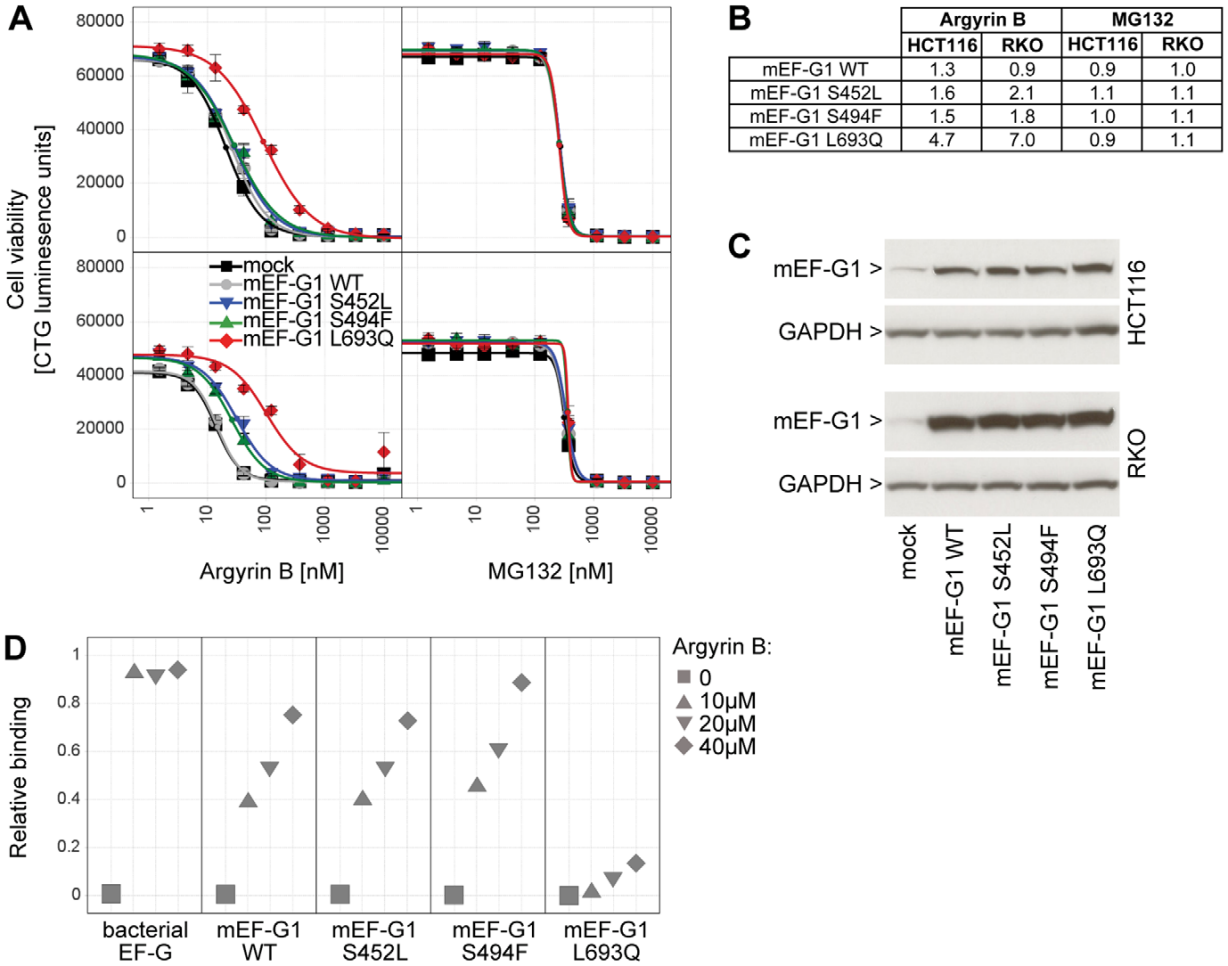
Rescue of argyrin B-sensitivity by expression of mEF-G1 L693Q. (**A**) mEF-G1 wild-type (WT), S452L, S494F or L693Q were stably over-expressed in HCT116 and RKO, cells were treated for 7 days with increasing doses of argyrin B or MG132, and cell viability was assessed using CellTiter Glo. A representative example of two independent experiments is shown. (**B**) Increase in IC_50_ relative to the parental cell line. Average fold increase was calculated from two independent experiments. (**C**) HCT116 and RKO stably expressing mEF-G1 WT, S452L, S494F or L693Q were lysed, and total proteins extracted and analyzed by immunoblotting for mEF-G1 and GAPDH. (**D**) Binding of argyrin B to recombinant human mEF-G1 WT, S452L, S494F or L693Q was measured by Biacore and is depicted relative to recombinant bacterial EF-G.

Given the anti-tumor activity of argyrins [6], [7], [8], [9] we next explored the argyrin B-sensitivity of mammalian cells by testing it at multiple doses across a panel of 512 human cancer cell lines by measuring ATP levels (CellTiter Glo) after a three day incubation. Argyrin B inhibited cell viability in 18 cell lines with an IC_50_ below 1 µM (**Figure 3A**). Comparing the IC_50_ and maximal activity profile of argyrin B to other cytotoxic agents revealed a strong correlation with another closely related natural argyrin (argyrin A), followed by mitochondrial electron transport inhibitors such as rotenone and antimycin A, but less so with other cytotoxic agents (**Figure 3B**). Electron transport complexes consist of several mitochondrial-encoded components, which are translated by mitochondrial ribosomes [21]. Hence, correlation of argyrin B to mitochondrial electron transport inhibitors may be caused by inhibition of mitochondrial protein synthesis via mitochondrial elongation factor G1 (mEF-G1, encoded by human *GFM1*), the closest mammalian homologue to bacterial EF-G and yeast mEF-G1 (**Table 2**). Supporting this, argyrin B treatment depleted mitochondrial-encoded cytochrome c oxidase subunit 2 (COX2), but did not affect the levels of nuclear-encoded succinate dehydrogenase flavoprotein subunit (SDHA) in two highly sensitive cell lines (HCT116 and RKO, **Figure 3C**). Furthermore, RKO cells became less sensitive to argyrin B under hypoxic culture conditions where energy metabolism switches from mitochondrial respiration to cytosolic glycolysis (data not shown). In line with mEF-G1 being the cellular target of argyrin B in mammalian cells, knock down of mEF-G1 by siRNA lowered the IC_50_ of argyrin B circa 9.6-fold in HCT116 and 3.3-fold in RKO cells without altering sensitivity to the proteasomal inhibitor MG132 (**Figure 3D and E**). The overall sequence homology between *P. aeruginosa* EF-G1 and human mEF-G1 is only 40%, but there is a striking conservation of amino acid residues shown to mediate binding to argyrin B and bacterial resistance (**Table 2**). We therefore introduced corresponding changes in human mEF-G1 (S452L, S494F, L693Q) to see if these reversed argyrin B-sensitivity in mammalian cells. Stable overexpression of mEF-G1 WT did not alter the argyrin B-sensitivity in HCT116 and RKO cells, and mEF-G1 S452L and S494F showed only marginal effects. However, expression of mEF-G1 L693Q decreased susceptibility to argyrin B by 4.7- and 7-fold in HCT116 and RKO cells, respectively, without altering sensitivity to MG132 (**Figure 4A, B and C**). It should be noted that the sensitive endogenous mEF-G1 is still expressed in these cells, which may decrease the magnitude of the susceptibility shift that can be mediated by the resistant mEF-G1 variants. Importantly, the rescue potential of the different mEF-G1 variants correlated well with their ability to bind argyrin B. Recombinant mEF-G1 WT, S452L and S494F still bound to argyrin B, with little binding detected with mEF-G1 L693Q (**Figure 4D**).

## Discussion

The data presented in this study strongly supports the notion that argyrin B inhibits bacterial protein synthesis and cell growth through binding of elongation factor G (EF-G) at a novel allosteric binding pocket. The detailed structural data elaborates a potential new mode of protein synthesis inhibition whereby EF-G (in complex with argyrin B) may be blocked from interacting with the ribosome. This may stand in contrast to the characterized inhibitor of EF-G, fusidic acid, which binds at the GDP/GTP binding pocket and interferes with the post translocation release of EF-G bound on the ribosome [22], [23], [24]. Supporting this mode of inhibition, argyrin B bound purified *P. aeruginosa* EF-G tightly, whereas fusidic acid binds EF-G in complex with the ribosome. Hence, argyrin B may constitute a new tool for the study of EF-G and ribosome function.

Argyrins also have an intriguing spectrum of antibacterial activity, which includes the Gram negative pathogen *P. aeruginosa*, for which a combination of membrane impermeability and active efflux mediates significant intrinsic resistance to antibacterial compounds. Although this activity is moderate (MIC = 4–8 µg/ml) it is nonetheless interesting since generally more susceptible Gram negatives such as *E. coli* and *S. typhimurium*, and even Gram positives such as *Staphylococcus aureus*, showed less susceptibility [2]. An *S. typhimurium* mutant defective in the outer membrane (increased permeability) was susceptible to argyrin, and these molecules inhibited in vitro transcription translation reactions, indicating that argyrins could inhibit those cells if there is sufficient cellular penetration. Consistent with this, we also observed substantially increased activity of argyrins against the efflux and membrane defective hypersusceptible *P. aeruginosa* mutant Z61. Intriguingly, we also demonstrated the previously unreported activity of argyrins against another notably intrinsically resistant Gram negative pathogen, *B. multivorans* (MIC = 1 µg/ml), whose EF-G is homologous to that of *P. aeruginosa* (Table 2). Based on these observations, the unique antibacterial spectrum of argyrins is determined in part by differences in cell penetration while other factors such as intrinsic target sequence variation likely also play a role, however this awaits further investigation.

In yeast and mammalian cells, argyrin B inhibited mitochondrial EF-G1, a close homologue of bacterial EF-G, consistent with the prokaryotic origin of mitochondria as predicted by the endosymbiosis theory [25]. The lack of inhibitory activity of argyrin B on glucose-grown yeast, which can utilize anaerobic fermentation, suggests an exclusive mitochondrial target in this context. We cannot exclude the possibility of additional targets of argyrin B in mammalian cells relating to the reported immunosuppressive activity [1], [2], [3], [4], [5], but our study identifies mEF-G1 as the major efficacy target for the suppression of mammalian cell growth. Since argyrin B-sensitive tumor lines showed an overall higher CellTiter Glow signal (data not shown), we hypothesize that susceptibility to argyrin B is dependent on carbon source utilization rates as well as the proliferative index, given that mitochondria need to divide and segregate as part of the cell division cycle. By inhibiting bacterial and mitochondrial EF-G, argyrin B blocks an important cellular machine, which is conserved in prokaryotic and eukaryotic cells, consistent with protein synthesis as a target of many natural products.

## Experimental Procedures

### Isolation of argyrin

Argyrins A and B were isolated from culture broths of *Actinoplanes sp.* 86317. The strain was cultivated in a medium consisting of potato starch 2.5%, glycerol 2.0%, soybean meal 0.5%, cornsteep powder 1.5%, yeast extract 0.3% and CaCO_3_ 0.5% for 7 days. Two liters of culture broth were filtered and mycelia were extracted with 1 L of ethyl acetate. The extract (2.7 g) was purified by reversed phase chromatography (RP18 as stationary phase) using formic acid (0.1%) and acetonitrile containing 0.1% formic acid as mobile phase yielding 5 mg argyrin B. The structures of both compounds were confirmed by comparison of the ^1^H-NMR data with the ^1^H-NMR published for Argyrin A and B [3], [5].

### Bacterial mutant selection experiments and *fusA1* sequencing

The strains used in this study are listed in Table 1. For isolation of mutants with decreased susceptibility to argyrin B, cultures were grown to mid-log phase (OD_600_, approximately 0.6–0.8) in Mueller-Hinton broth, collected by centrifugation, and resuspended in fresh medium and plated on Mueller-Hinton agar containing argyrin B. Serial dilutions were also plated on Mueller-Hinton agar without compound for enumeration (colony forming units/ml, CFU/ml). Resistance frequencies were calculated as the number of CFU (mutants) on drug containing plates divided by the number of CFU plated. Whole genome sequencing was done as described previously [26]. The *fusA1* gene from *P. aeruginosa* was generated for sequencing in two parts, using primer pairs PAEFF1/PAEFR1: GGCCATGCGTTGGCTGGTGGAC/GTGACGTCCTTCATGCCGATC and EFF2/EFR1: GTTCAAGAACAAGGGCGTTC/GGTGCCGACGTTGACGTGCGG. The *fusA1* gene from *B. multivorans* was generated as a single band using BCEFGF1/BCEFGR1: CATTTCCGTTTCTAAGCGCC/CAATCGTACCAACGTTCACGTG. Nucleotide sequencing of the products was done by Agencourt (Beverly MA).

### Purification of *P. aeruginosa* EF-G1 and EF-G1_S459F_

For wild type *P. aeruginosa* EF-G1 expression, the *fusA* gene was amplified from genomic DNA of *P. aeruginosa* PAO1 K767 using the primer pair EFG-F/EFG-R: AGC*CATATG*GCCCGTACTACACCCATCAACC/AAA*AAGCTT*ATCAACCTTGTTTTTTAACCAGCGC. The product was digested with *Nde*I and *Hind*III and ligated into similarly digested pET28a to generate pET28a-PaEFG-Nhis. This plasmid was then used for site directed mutagenesis to introduce the nucleotide change encoding EF-G1_S459F_ (pET28a-PaEFGS459F-Nhis). The plasmids were then transformed into an *E. coli* strain BL21 (DE3) for expression. A single colony was used to inoculate 10 ml LB broth containing 50 µg/ml kanamycin which was incubated at 37°C overnight, with shaking at 250 rpm. The overnight culture was then diluted 100-fold into 1 liter fresh LB broth containing 50 µg/ml kanamycin. After growing at 37°C with vigorous shaking for about 2.5 hours, expression of *P. aeruginosa* EF-G1 or EF-G1_S459F_ was then induced by adding 0.1 mM IPTG, with further culture incubation at 18°C for 24 hours. Cells were then pelleted by centrifugation at 5,000 rpm with Sorvall SLC-6000 rotor and stored at −20°C. Cell pellets were then resuspended in 20 ml of BugBuster solution (Novagen) and incubated at room temperature for 20 minutes. The cell lysate was centrifuged at 20,000 rpm in a Sorvall SA-300 rotor for 20 min at 4°C. The supernatant was then loaded onto a Ni-NTA agarose column with bed volume of 2.5 mL. The mixture in the column was then rotated on a platform mixer at 4°C for an hour. The column was washed with 10 column volumes (CV) of wash buffer (100 mM Tris-HCl pH 8, 200 mM NaCl, 10 mM imidazole), followed by 5 CV of wash buffer containing 40 mM imidazole. The bound his-tagged *P. aeruginosa* EF-G1 proteins were eluted with 5 CV of buffer containing 500 mM imidazole. The eluent was concentrated to about 2 ml using an Ultracel-30K centrifugal filter unit with a molecular weight cutoff of 30 kDa (Amicon). The resulting protein solution was centrifuged at 13,000 rpm at 4°C with a bench top centrifuge, and then filtered with an Anotop 10 filter to remove any possible large aggregates before being injected onto a HiLoad 16/60 Superdex 200 SEC column and purified by AKTA FPLC system. The chromatography was conducted using running buffer containing 50 mM Tris-HCl, pH 7.5, 10 mM MgCl_2_, 1 mM DTT, 50 mM NH_4_Cl, 10% Glycerol at a flow rate of 1 mL/min. Fractions of 2.5 mL each were collected. The protein in the fractions was monitored by UV absorbance at 280 nm. Fractions containing *P. aeruginosa* EF-G1 protein were pooled and stored at −80°C. Protein concentration was determined by Nano-drop spectrophotometer. The identity of *P. aeruginosa* EF-G1 proteins was confirmed by SDS-PAGE and molecular weight determination using an LC-ESI-MS system (Agilent 1100/Waters ZQ4000).

### Isothermal titration calorimetry (ITC) determinations

For isothermal titration calorimetry, *P. aeruginosa* EF-G1 was dialyzed against 50 mM Tris-HCl pH 7.6, 10 mM MgCl_2_, 1 mM TCEP, 10 mM NH_4_Cl, 100 µM GDP, in a 3 mL dialysis cassette (10K MWCO) at 4°C overnight. DMSO and buffer were used to adjust to 44 µM EF-G1 and 2% DMSO in 0.5 mL. Argyrin B was diluted to a final concentration of 2.5 µM (2.0 mL) with the dialysis buffer and DMSO (2% final concentration). Both protein and compound solutions were degassed and loaded onto the VP-ITC titration calorimetry instrument according to the manufacturer's instruction. After initial injection of 2 µL, *P. aeruginosa* EF-G1 was injected into the cell containing argyrin B at the volume of 10 µL per injection with interval of 240 seconds. The stirring rate was 307 rpm and the titration temperature was 25°C. The data was analyzed using nonlinear least-squares curve fitting in MicroCal Origin Version 7.0. The standard one binding site model was used to obtain the thermodynamic parameters K (binding constant), enthalpy of binding (ΔH), entropy of binding (ΔS), and n, where n is the ratio of argyrin B to *P. aeruginosa* EF-G1 in the complex.

### Surface plasmon resonance (SPR) determinations

EF-G1 proteins (0.5 ml) were first dialyzed against 1 L 1× PBS using the 10K MWC Slide-A-Lyzer 10K MWCO at 4°C overnight. EZLink Sulfo-NHS-LC-LC-Biotin (10 mM in PBS) was added to FusA1 to provide a molar ratio of crosslinking reagent and *P. aeruginosa* EF-G1 of about 1∶0.5 to 1∶1. This mixture was incubated overnight at 4°C. Unreacted and hydrolyzed biotin reagent was removed with a Zeba desalting chromatography cartridge by following the manufactures instructions. Labelling with a single biotin was then confirmed by LC-ESI-MS. Biacore running buffer contained 50 mM Hepes, pH 7.4, 0.25 M NaCl, 10 mM MgCl_2_, 1 mM TCEP, 2% DMSO and 0.05% surfactant P20. The SA chip was activated with three consecutive 1 min injections at 10 µl/min of pre-conditioning solution (1 M NaCl and 40 mM NaOH) prior to the immobilization. A “Prime” procedure was performed to flush the chip free of the pre-conditioning solution. Biotinylated EF-G1 was immobilized on the sample flow cells on SA chip with a flow rate of 10 µl/min to about 2,000 response units (RU). The unoccupied biotin binding sites on the SA chip were blocked by injecting 10 mM Biotin over all sample and reference flow cells for 3 mins at 10 µl/min. Compounds were prepared with different dilutions (1.53 nM, 4.6 nM, 13.7 nM, 41.2 nM, 123 nM, 370.4 nM, 1,111.1 nM, 3,333.3 nM and 10,000 nM) on a 96 or 384 plate. The final concentration of DMSO was 2%. Binding of the compounds to the immobilized EF-G1 was done by running at 50 µl/min with 200 seconds of association time and 250 seconds of dissociation time. Data was analyzed by global fitting into a 1∶1 binding model using the Biacore T100 Evaluation Software Version 2.0 and the association rate constant (*k*_a_), dissociation rate constant (*k*_d_), and dissociation constant (*K*_D_ = *k*_d_/*k*_a_) were obtained as fitting results. If the binding reaches equilibrium at the end of association phase of each concentration injection, the *K*_D_ value can also be determined by measuring the average data points (over 5 seconds) near the end of the association phase (4 seconds before the end of injection). The obtained RU is then plotted against the compound concentration and fitted to the steady-state affinity model (Langmuir equation) using the Biacore T100 Evaluation Software Version 2.0 to calculate the *K*_D_.

### Crystallography of the EF-G argyrin complex

BL21 (DE3) cells harboring pET28a-PaEFG-Nhis were grown in Terrific Broth containing 100 µg/ml kanamycin. EF-G1 expression was induced using 1 mM IPTG. Cell pellets were resuspended and lysed using a microfluidizer, after which ultracentrifugation was performed. Purification was done using NiNTA resin followed by removal of the N-terminal Histidine tag with thrombin protease. The protein was further purified using a Mono Q column and was then concentrated and loaded onto a HiLoad Superdex 200 26/60 column, exchanging the protein into 25 mM Tris-HCl pH 7.5, 150 mM NaCl and 1 mM TCEP. For crystallization of the protein with argyrin B, 2 mg/ml *P. aeroginosa* EF-G1 was incubated with 200 µM argyrin B for 1 hour on ice followed by concentration of the protein to 11 mg/ml. Sitting drop vapor diffusion method was used for crystallization, with the crystallization well containing 100 mM Tris-HCl pH 7.5, 18% PEG 3350 and 200 mM sodium nitrate, and the drop containing a 1∶1 volume of protein and crystallization solution. After crystals formed they were subsequently cryoprotected using the crystallization solution with the addition of 20% glycerol, followed by flash freezing directly into liquid nitrogen.

### Data collection and structure determination

Diffraction data for the *P. aeroginosa* EF-G1/argyrin B complex were collected on a Dectris Pilatus 6M Detector at the Advanced Photon Source beamline 17-ID at a wavelength of 1 Å. The data were measured from a single crystal maintained at 100°K, and the reflections were indexed, integrated, and scaled using autoPROC [27], [28]. The spacegroup of the complex is C2 with 1 molecule in the asymmetric unit. The structure was determined with PHASER [29] using molecular replacement methods with a starting model of *Thermus thermophilus* EF-G (PDB Code 2BM0) [30]. The starting model was broken into three search models, which included Domains I and II, Domain IV, and Domain V while Domain III was fit manually. Structure determination was achieved through iterative rounds of positional and simulated annealing refinement using BUSTER (BUSTER, version 2.8.0. Cambridge, United Kingdom: Global Phasing Ltd.), with model building using COOT [31]. Individual B-factors were refined using an overall anisotropic B-factor refinement along with bulk solvent correction. The argyrin B as well as the solvent molecules were built into the density in later rounds of the refinement. Data collection and refinement statistics are shown in Table 3. The structure of *P. aeruginosa* EF-G1 in complex with argyrin B contains protein residues Met1-Leu40, Thr62-Asn194, Lys198-Arg408, Phe411-Pro422, Leu435-Val479, Ile483-Gln705, 1 molecule of Argyrin B and 21 solvent molecules. The coordinates and structure factors have been deposited in the Protein Data Bank (PDB ID Code: 4FN5).

**Table 3:** Crystallographic data and refinement information.

parameters		*pa*EF-G/argyrin B complex
space group		C2
		a = 125.8 Å
		b = 88.1 Å
		c = 74.3 Å
		α = γ = 90°
		β = 107.9°
resolution range (Å)		20.0-2.9
total observations		56696
unique reflections		16969
completeness (%)^a^		98.7 (99.9)
*I/σ* a		13.5 (2.0)
*R*_sym_ a ^*,*^ b		0.061 (0.500)
*R*_cryst_/*R*_free_ c		0.242/0.337
protein atoms		5177
heterogen atoms		76
solvent molecules		16
Average *B*-factor (Å^2^)		84.5
	rms deviations from ideal values	
bond lengths (Å)		0.01
bond angle (°)		1.30

a Numbers in parenthesis are for the highest resolution shell (3.06-2.90).

b R_sym_ = Σ|I_h_−<I_h_>|/ΣI_h_ over all h, where I_h_ is the intensity of reflection h.

c R_cryst_ and R_free_ = Σ∥F_o_|−|F_c_∥/Σ|F_o_|, where F_o_ and F_c_ are observed and calculated amplitudes, respectively. Rfree was calculated using 5% of data excluded from the refinement.

### Yeast susceptibility determination

Yeast strains used in this study are BY4741 [32] and strain BYΔ8, derived from BY4741 but deleted for eight genes involved in drug resistance (efflux pumps: *snq2*, *pdr5*, *yor1*; transcription factors: *pdr1*, *pdr2*, *pdr3*, *yap1*, *yrm1*). Argyrin potency was determined as follows: 11 point serial dilutions (3.1 dilution factor) were prepared in 96 well plates with log phase growth yeast cultures in YPD (2% glucose, 2% BactoPeptone, 1% yeast extract) and YPEG (2% glycerol, 1% ethanol, 2% BactoPeptone, 1% yeast extract), giving a compound dilution range from 200 µM to 2.44 nM and a DMSO control. Starting OD_600_ was 0.1, total volume per well 120 µl. Plates were incubated at 30°C with 770 RPM orbital shaking. The 16 h time point in YPD and the 42 h time point in YPEG represented late log phase and was used to calculate IC_50_ values using a logistic regression curve fit algorithm.

### Microscopy of mitochondrial morphology

BY4741 cells containing a genomic COX4-GFP fusion where grown to mid-log phase in glucose and ethanol/glycerol media and incubated with the corresponding concentrations of compound for 30 minutes. Aliquots where spotted on microscopy slides and directly imaged on a Zeiss Axiovert 200Mot microscope equipped with a AxioCam MRm digital camera using the Plan-Neofluar 100×, 1.3 oil PH3 objective (Carl Zeiss, Feldbach, Switzerland). A z-stack consisting of 3 images spaced by 0.75 µM in the filterset 38 (eGFP) channel and a phase-contrast image where acquired. False colors where applied to the GFP images (green) and phase contrast (blue) and the images merged into one plane and scaled to 8-bit. Representative, individual cells where selected and assembled into a panel using Adobe Photoshop (Adobe Systems Inc.).

### Selection of drug resistant *S. cerevisiae* cells

Strain BYΔ8 was incubated in 2.5% ethylmethanesulfonate until only 50% of the cells formed colonies. A total of 2×10^7^ mutagenized BYΔ8 cells were plated on two 14 cm dishes with synthetic complete medium (0.7 g/l Difco Yeast Nitrogen Base w/o amino acids, 0.79 g/l MPbio CSM amino acid mixture, 2% Glycerol, 1% Ethanol) containing 0.1 µM Argyrin B. After 3 days resistant colonies appeared. Resistance was confirmed by re-streaking on 0.1 µM Argyrin B. Genomic DNA was extracted (YeaStar Genomic DNA Extraction Kit, Zymo Research) and the genome analyzed by 80 nucleotide paired end reads on an Illumina HighSeq 2000. Analysis of the obtained sequence showed an average of 180× coverage. Amplifying the mutated *MEF1* gene and transforming it into non-mutagenized BYΔ8 cells validated identified mutations in *MEF1*. Integration by homologous recombination was selected for on plates containing 0.1 µM argyrin B. Presence of the corresponding point mutations were confirmed by direct sequencing of the *MEF1* gene. Resistance was confirmed by recording dose-response curves in with serial dilutions of argyrin B as described above.

### Mammalian cell line profiling and compound correlation analysis

A panel of 512 human cancer cell lines (http://www.broadinstitute.org/ccle) was tested for sensitivity to various pharmacological agents, including argyrin A, argyrin B, rotenone, antimycin A, topotecan, paclitaxel, 17-AAG, staurosporin and velcade. All cell lines were maintained in humidified incubators at 37°C and 5% CO_2_ in DMEM or RPMI1640 supplemented with 10% FBS. Cells were seeded into 1536-well plates at a density of 250 cells per well, allowed to adhere for 12–24 hours, then treated for 3 days with pharmacological agents in 8-point dose response (semi-log from 8 µM to 2.5 nM), and cell viability was assessed using CellTiter Glo (Promega). Automatic curve fit was used to determine IC_50_ and maximal activity (A_max_) values, relative to 0.4% DMSO (0%) and 1 µM MG132 (−100%) [33]. Compound correlation analysis was performed by calculating a Pearson correlation for each compound pair (using either IC_50_ or A_max_ values) across the 512 cell lines. We required data values for a minimum of 50 cell lines in common to avoid spuriously high correlations.

### Cell viability assays

HCT116 (ATCC, CCL-247) and RKO (ATCC, CRL-2577) cell lines were maintained in a humidified incubator at 37°C and 5% CO_2_ in RPMI1640 supplemented with 10% FBS. Cell viability was determined using the CellTiter-Glo luminescent cell viability assay (Promega). 1000 cells were seeded into wells of clear-bottom 384-well plates in 30 µl growth media and treated for 7 days with dose responses of argyrin B or MG132 (Sigma) by adding 10 µl compound-containing media per well. Subsequently, 40 µl of CellTiter-Glo reagent was added to each well and luminescence recorded on an Envision plate reader (Perkin Elmer). Each treatment condition was tested in triplicate and compared to an equivalent volume of DMSO as control.

### siRNA transfection

Non-targeting (NT) or GFM1 siRNA pools were obtained from Dharmacon (ON-TARGETplus SMART pool D-001810 and L-013685). HCT116 and RKO cells were reverse transfected in 6-well plates using RNAiMax (Invitrogen) and 25 nM siRNA. After 2 days, cells were harvested by trypsinization and re-plated into 6-well plates for western blot analysis or into 384-well plates for compound treatments and cell viability determination using CellTiter-Glo as described above.

### Mutagenesis of mEF-G1

The human GFM1 cDNA (encoding mEF-G1, Table 2) was mutagenized in pENTR221 (Invitrogen) using QuikChangeII XL site-directed mutagenesis (Stratagene). The following point mutations were introduced: C1355T (encoding S452L), C1481T (encoding S494F) and T2078A (encoding L693Q). The GFM1 cDNAs were then subcloned into the mammalian expression vector pLenti4/TO/V5 using LR clonase II (Invitrogen) and sequence-verified. The pLenti4 constructs were then co-transfected with a lentiviral plasmid mix (encoding LP1, LP2 and VSV-G) into 293T cells, and viral particles were harvested after 72 h and used to infect HCT116 and RKO cells. Stable cell lines were generated by selection with 400 µg/ml Zeocin and plated into 6-well plates for western blot analysis or into 384-well plates for compound treatments and cell viability determination using CellTiter-Glo as described above.

### Immunoblotting for determination of mitochondrial protein synthesis inhibition

Whole cell lysates were prepared using RIPA buffer (Cell Signaling Technology) supplemented with EDTA-free protease inhibitor cocktail (Roche Applied Science), cleared by centrifugation at 16,000× g for 10 min, normalized based on protein concentration (Bio-Rad protein assay), boiled in NuPAGE LDS sample buffer (Invitrogen) supplemented with 2% ß-mercaptoethanol, and separated on NuPAGE 4–12% Bis-Tris gels using MOPS running buffer (Invitrogen). Proteins were transferred to nitrocellulose membranes and probed with the following primary antibodies: rabbit polyclonal antibody against mEF-G1 (Sigma, HPA034765), rabbit monoclonal antibody against GAPDH (Cell Signaling Technology, 14C10), mouse monoclonal antibodies against COX2 (Invitrogen, A6404) or SDHA (MitoSciences, MS204). Immobilized primary antibodies were visualized using HRP-conjugated anti-rabbit or anti-mouse IgG (Millipore, AP307P and AP308P) and enhanced chemiluminescence (Pierce).

### Human EF-G1 protein production and surface Plasmon Resonance

GFM1 encoding the active site of EF-G1 (residues 36–751) was PCR amplified from pDONR221 constructs using primers (GGG GAC AAG TTT GTA CAA AAA AGC AGG CTT AAT GTC TTC ATC AGG GGT GAT T, GGG GAC CAC TTT GTA CAA GAA AGC TGG GTA TTA GTT CTT GGC TTT TCC). *P. aeroginosa fusA1* encoding full-length EF-G was PCR amplified from pET28a-PaEFG-Nhis using primers (GGG GAC CAC TTT GTA CAA GAA AGC TGG GTA TTA TCA ACC TTG TTT TTT, GGG GAC AAG TTT GTA CAA AAA AGC AGG CTT AAT GGC CCG TAC TAC ACC C). Purified PCR products were cloned into the pDONR221 vector using Gateway cloning and BP Clonase II (Invitrogen), shuttled into an *E.coli* protein expression vector for N-terminal tagging (Avitag-His6 vector) using LR Clonase II (Invitrogen), and verified by sequencing. Avitag-His6-hEF-G1 and Avitag-His6-PaEF-G constructs were transformed into BL21-AI cells (Invitrogen) and isolated single colonies were used to inoculate 5 ml cultures which were grown overnight at 37°C with shaking. Two milliliters of the overnight cultures were then used to inoculate 1 liter of TB, grown at 37°C until the OD_600_ reached 0.4, adjusted to 0.2% arabinose, and incubated overnight at 18°C. Subsequently, cells were pelleted, lysed in 30 mls of Q-proteome buffer (Qiagen) supplemented with EDTA-free protease inhibitor cocktail (Roche), and lysates were centrifuged at 24,000× g for 30 minutes and further clarified by passage through a 0.45 µm filter. hEF-G1 and PaEF-G protein were purified using 1 ml HisTrap FF column on AktaXpress (GE Healthcare). Briefly, protein was captured on column using binding buffer (50 mM Tris; pH 7.5, 300 mM NaCl, 20 mM imidazole, 10% glycerol), washed extensively with washing buffer (50 mM Tris; pH 7.5, 500 mM NaCl, 30 mM imidazole, 10% glycerol), and then eluted with elution buffer (50 mM Tris; pH 7.5, 500 mM NaCl, 250 mM imidazole, 10% glycerol). Peak protein fractions were pooled and further purified using a size exclusion column (16/60 Superdex column) equilibrated in 50 mM Tris; pH 7.5, 500 mM NaCl, 10% glycerol, and stored at −80°C. For Biacore, a CM5 chip was activated with NHS-EDC, and neutravidin was immobilized to the surface using 50 µg/ml neutravidin (Pierce) in acetate buffer; pH 4.5. 7000 RU's of each protein was bound to the chip surface in HBS-P+ buffer (10 mM HEPES; pH 7.4, 150 mM NaCl, 0.05% surfactant P20). Argyrin B was added to HBS-P+ buffer at 10, 20, and 40 µM final concentrations in 4% DMSO, and profiled using 120 second contact time, 60 second dissociation with a 30 µl/min flow rate in running buffer (HBS-P+ buffer containing 4% DMSO). All data was solvent corrected.
